# Providence for Drunkards

**Published:** 1884-12

**Authors:** 


**The Providence for Drunkards.**—Drunkards enjoy a remarkable immunity from the consequences of injuries. One sometimes sees a drunken man pitched violently from a horse, and when the by-standers rush to the spot, expecting to find him dead, they are astonished to discover that he has been little injured. In his "Scrambles Among the High Alps," Leslie Stephen tells the story of a guide who while drunk fell over a precipice so deep that a fall over it seemed almost certain death, and yet he sustained little injury. Stephen accordingly gives his readers the advice either not to fall over a precipice, or to get thoroughly drunk before doing so. A man has thrown himself while drunk over the Dean bridge, in Edinburg, a height of about 200 feet, on to the rocky bed of the stream below. A sober man would probably been instantly killed, but this individual, though he broke both of his thigh bones, recovered. The reason of this immunity probably is that the nerve centres, which regulate the heart and vessels, are so paralyzed in the drunken man as not to be affected by the shock of the fall, which in a sober man would have acted on them so violently as to stop the heart, arrest the circulation, and cause instant death.—*Medical Journal.*

